# ERRATUM: Adherence to central venous catheter insertion bundle in neonatal and pediatric units

**DOI:** 10.1590/1980-220X-REEUSP-2024-ER23en

**Published:** 2025-01-27

In the article “Adherence to central venous catheter insertion bundle in neonatal and pediatric units”, with DOI number: http://dx.doi.org/10.1590/S1980-220X2017009603269, published in the Revista da Escola de Enfermagem da USP, volume 2017;51:e03269 of 2017, p.1–7, on page 7:

Where it was written:

## Financial support

Conselho Nacional de Desenvolvimento Científico e Tecnológico – CNPq. Process no. 443997/2014-4.

Now read:

## Financial support

Conselho Nacional de Desenvolvimento Científico e Tecnológico – CNPq. Processo n. 443997/2014-4.

Fundação de Amparo à Pesquisa do Estado de Minas Gerais (FAPEMIG) APQ-02531-17.

