# Supplementary material for: ERRATUM: Adherence to central venous catheter insertion bundle in neonatal and pediatric units
Source: Rev Esc Enferm USP. 2025 Jan 27;58:e2024ER23. doi: 10.1590/1980-220X-REEUSP-2024-ER23en (PMC11771850; doi:10.1590/1980-220X-REEUSP-2024-ER23en)
Supplement: Translation

# Translation: ERRATA: Adhesión al bundle de inserción de catéter venoso central en unidades neonatales y pediátricas

**DOI:** 10.1590/1980-220X-REEUSP-2024-ER23es

En el artículo “Adhesión al bundle de inserción de catéter venoso central en unidades neonatales y pediátricas”, con el número DOI http://dx.doi.org/10.1590/S1980-220X2017009603269, publicado en la Revista da Escola de Enfermagem da USP, volume 2017;51:e03269 de 2017, p. 1-7, en la página 7:

Donde se leía:

## Apoyo financeiro

Conselho Nacional de Desenvolvimento Científico e Tecnológico – CNPq. Expediente n. 443997/2014-4.

Lea:

## Apoyo financeiro

Conselho Nacional de Desenvolvimento Científico e Tecnológico – CNPq. Expediente n. 443997/2014-4.

Fundação de Amparo à Pesquisa do Estado de Minas Gerais (FAPEMIG) APQ-02531-17.

# Translation: ERRATA: Adesão ao bundle de inserção de cateter venoso central em unidades neonatais e pediátricas

**DOI:** 10.1590/1980-220X-REEUSP-2024-ER23pt

No artigo “Adesão ao bundle de inserção de cateter venoso central em unidades neonatais e pediátricas”, com número de DOI: http://dx.doi.org/10.1590/S1980-220X2017009603269, publicado na Revista da Escola de Enfermagem da USP, volume 2017;51:e03269 de 2017, p.1-7, na página 7:

Onde se lê:

## Apoio Financeiro

Conselho Nacional de Desenvolvimento Científico e Tecnológico – CNPq. Processo n. 443997/2014-4.

Leia-se:

## Apoio Financeiro

Conselho Nacional de Desenvolvimento Científico e Tecnológico – CNPq. Processo n. 443997/2014-4.

Fundação de Amparo à Pesquisa do Estado de Minas Gerais (FAPEMIG) APQ-02531-17.
